# Reduced miR-181d level in obesity and its role in lipid metabolism via regulation of ANGPTL3

**DOI:** 10.1038/s41598-019-48371-2

**Published:** 2019-08-14

**Authors:** Mohamed Abu-Farha, Preethi Cherian, Irina Al-Khairi, Rasheeba Nizam, Abdullah Alkandari, Hossein Arefanian, Jaakko Tuomilehto, Fahd Al-Mulla, Jehad Abubaker

**Affiliations:** 1 Biochemistry and Molecular Biology Unit, Dasman Diabetes Institute, Al Kuwayt, Kuwait; 2 Functional Genomic Unit, Dasman Diabetes Institute, Al Kuwayt, Kuwait; 3 Clinical Trial Units, Dasman Diabetes Institute, Al Kuwayt, Kuwait; 4 Immunology Unit, Dasman Diabetes Institute, Al Kuwayt, Kuwait; 5 Research Division, Dasman Diabetes Institute, Al Kuwayt, Kuwait

**Keywords:** Prognostic markers, Type 2 diabetes

## Abstract

Obesity impacts the endocrine and metabolic functions of the adipose tissue. There is increasing interest in the role of epigenetic factors in obesity and its impact on diabetes and dyslipidemia. One such substance, miR-181, reduces plasma triglyceride levels in mice by targeting isocitrate dehydrogenase 1. In the other hand, the adipocyte differentiation and lipid regulating hormone angiopoietin-like 3 (ANGPTL3) is a known regulator of circulating apolipoproteins through its inhibition of the lipoprotein lipase activity. We aimed to study the miR-181d expression in the blood and adipose tissue in a cohort of obese and non-obese people, assessing its possible role in obesity. We also aimed to confirm whether miR-181d can bind and regulate ANGPTL3. miR-181d expression levels were investigated in 144 participants, 82 who were non-obese (body mass index [BMI] < 30) and 62 who were obese (BMI > 30). miR-181d levels in plasma and adipose tissue were measured by RT-PCR. Hepatocyte cell cultures were assessed by overexpression and 3′-UTR-luciferase assays for miR-181d binding to its target protein and its effect on the protein. The plasma levels of ANGPTL3 were also measured by ELISA. The miR-181d levels were significantly lower in obese than in non-obese individuals. *In vitro* analysis confirmed miR-181 binding to and repression of the ANGPTL3 transcript. Obesity leads to alterations in miR-181d expression. Its downregulation in obese humans was inversely correlated with ANGPTL3, a protein involved in adipocyte differentiation and lipid metabolism. miR-181d can be used as an inhibitor of ANGPTL3 to reduce the TG plasma level.

## Introduction

Obesity is a global epidemic and an increasing concern worldwide for peoples’ health and well-being. Its rising prevalence is due to increased energy intake and a sedentary lifestyle^1^. Obesity predisposes individuals to numerous health complications such as type 2 diabetes, hypertension and cardiovascular disease. Obesity is associated with excessive lipid accumulation as well as dysregulation of the lipid metabolism pathways that control lipid synthesis and oxidation^2^. Numerous reports have shed light on the role of microRNAs in regulating many biological processes associated with obesity and diabetes, such as adipocyte differentiation and lipid metabolism^3–5^. For example, miR-122, an miRNA abundant in the liver, has been shown to modulate cholesterol synthesis and fatty acid oxidation by regulating FAS (fatty acid synthase), ACC1 and ACC2 (Acetyl-CoA carboxylases 1 and 2) proteins^6^. miR-33 embedded within SREBF1 genes is a main regulator of lipid metabolism, since it downregulates several ABC transporters such as ABCA1 and ABCG1, thereby regulating cholesterol and high-density lipoprotein (HDL) production^7^. Moreover, miR-33 has been linked to fatty acid degradation and the macrophage response to low-density lipoprotein (LDL)^8^. An earlier report showed that miR-181d was the most efficacious inhibitor of hepatic lipid droplets, decreasing them by about 60%^9^. Additionally, miR-181d reduces cellular triglycerides (TG) and cholesterol ester through its inhibition of isocitrate dehydrogenase 1 (IDH1)^10^.

One of the key enzymes involved in regulating plasma TG level is lipoprotein lipase (LPL)^11–14^. It is responsible for the hydrolysis of TG-rich lipoprotein into fatty acids that can be utilised by peripheral tissues or stored in adipocytes. LPL enzymatic activity is regulated by various factors, including the angiopoietin-like proteins ANGPTL3, 4 and 8^15–17^. These proteins belong to a family of eight members (ANGPTL1–8) that are associated with various metabolic pathways, including insulin resistance, oxidative stress and dyslipidemia^18^. ANGPTL3 regulates the activity of LPL through its interaction with ANGPTL8^18,19^. Following its production in the liver, ANGPTL3 is proteolytically cleaved by proprotein convertases to generate an active N-terminal domain that inhibits LPL activity^20–22^. Earlier studies showed that loss of function mutations identified in ANGPTL3 was associated with reduced levels of very low-density lipoprotein (VLDL), LDL, HDL and TG^23^. Our recent study showed increased ANGPTL3 levels in obese humans without diabetes as compared with healthy normal-weight controls^24^.

ANGPTL3 inhibitors are pursued as potential therapeutic agents to reduce lipid levels in plasma. We designed this study to identify microRNAs that can inhibit the activity of ANGPTL3. The Bioinformatic analysis suggested miR-181d as one such agent. miR-181d has two putative binding sequences within the 3′UTR of human ANGPTL3 that can possibly confer inhibition of translation of ANGPTL3 gene. Our Hypothesis was that miR-181d is likely to bind and repress ANGPTL3 and reverse its LPL inhibition and improve dyslipidemia. This study was designed to examine the miR-181d expression levels in obesity and to investigate its association with ANGPTL3. We assessed the interaction between miR-181 and ANGPTL3 using a luciferase assay. We also studied the impact of its overexpression and its mimics on the expression of ANGPTL3 in a hepatocyte cell model under normal conditions and after treatment with free fatty acids.

## Methods

### Study population

The study was conducted among two groups of adult men and women who were either non-obese (body mass index [BMI] = 20–29.9 kg/m^2^) or obese (BMI = 30–40 kg/m^2^) as described previously^24–27^. Informed written consent was obtained from all individuals for their participation in the study. The study was approved by the Review Board of Dasman Diabetes Institute and carried out in line with the ethical guideline declaration of Helsinki. Exclusion criteria included morbid obesity (i.e. BMI > 40 kg/m^2^), prior major illness or use of medications or supplements known to influence body composition or bone mass.

### Anthropometric and biochemical measurements

Plasma was prepared from peripheral blood samples from all subjects using vacutainer EDTA tubes and then aliquoted and stored at −80 °C until assayed as described previously^28–30^. Adipose tissue biopsies (about 1 g) were obtained from 10 subjects in each group from the periumbilical area by surgical biopsy after local anaesthesia as described previously^28^. The biopsy specimen was rinsed in cold phosphate-buffered saline, divided into four pieces and stored at −80 °C until assayed. The average of three blood pressure readings measured using an Omron HEM-907XL Digital sphygmomanometer was taken, with a 5–10 min rest between each reading. Whole-body composition was determined using a dual-energy radiographic absorptiometry device (Lunar DPX, Lunar radiation, Madison, WI). Fasting blood glucose, TG, total cholesterol, LDL and HDL were measured on the Siemens Dimension RXL chemistry analyser (Diamond Diagnostics, Holliston, MA). Glycated haemoglobin (HbA1C) was determined using the Variant device (Bio-Rad, Hercules, CA).

### RNA extraction and reverse transcription from adipose tissue

Subcutaneous adipose tissue samples were stored immediately after biopsy in 1 mL RNAlater-RNA Stabilisation Reagent (Qiagen Inc., Valencia, CA) at −80 °C. Total RNA, including microRNA, was extracted using miRNeasy Kits (Qiagen Inc.). miRNA was converted to cDNA using the miScript II RT kit (Qiagen Inc.). RT-PCR for the detection of mature miRNA was performed using an miScript SYBR Green PCR Kit. The cycling conditions for the RT-PCR were as follows: the initial activation step for 15 min at 95 °C, during which the HotStarTaq DNA Polymerase was activated and 40 cycles of three-step cycling which included denaturation at 94 °C for 15 s, annealing at 55 °C for 30 s and extension at 70 °C for 30 s. Fluorescence data collection was carried out to detect the CT value, and relative expression was assessed using the ∆∆CT method^31^ after normalisation with RNU6B and RNU48 RNA. (Table 1 shows the primer sequences).

**Table 1:** Nucleic acid sequences for miRNAs and mimics.

Primer name	Sequence	Company
Hs_miR-181d_2	5′AACAUUCAUUGUUGUCGGUGGGU	MS00031500– Qiagen
Syn-has-miR-181 mimic	5′‘AACAUUCAUUGUUGUCGGUGGGU	MSY0002821- Qiagen
RNU6B	5′-TGACACGCAAATTCGTGAAG-3′	Qiagen
RNU48	5′-CTGCGGTGATGGCATCAG-3′	Qiagen

### Bioinformatic prediction of miRNAs

Targetscan (http://www.targetscan.org/) and miRWalk (http://mirwalk.umm.uni-heidelberg.de/) prediction softwares were used to identify the miRNAs that can target ANGPTL3 gene with the highest probability, which allowed us to aggregate and compare results from other mRNA-to-miRNA databases. These web sites predict biological targets of miRNAs by searching for the presence of conserved 8mer, 7mer, and 6mer sites that match the seed region of each miRNA. For sites matching highly conserved miRNA families, there are two complementary choices, looking at either site conservation or the predicted efficacy of the site. smIR-181d was identified as a possible miRNA to target ANGPTL3 gene with two binding sites.

### microRNA extraction from plasma

For the extraction of microRNA from plasma, the samples were centrifuged at 16,000 g × 10 min at 4 °C (a step recommended by the protocol to eliminate residual cellular material) and extracted using ExoRNeasy Serum/Plasma Midi Kit (Qiagen). The extracted RNA was then converted to cDNA using miScript RT kit (Qiagen). As per the kit protocol, 3.5 µl (1.6 × 108 copies/µl working solution) of miRNeasy serum/ plasma spike-in control (Qiagen, MD, USA) was added to each sample while processing. Furthermore, the exoRNeasy Serum/Plasma Kits include a miScript Primer Assay that detects the miRNeasy Serum/Plasma Spike-In Control using real-time PCR. This was used to normalize for miRNA expression in plasma. The cDNA was diluted as recommended by the kit protocol and used for gene expression studies. SNORD61 RNA was used as a normaliser in the gene expression analysis (cat. MS00033740, Qiagen), and the ∆∆CT method was used to assess the expression of miR-181d (cat. MS00031500, Qiagen, Table 1) in the plasma.

### ANGPTL3 enzyme-linked immunosorbent assay

The plasma levels of ANGPTL3 were assessed using a multiplexing immunobead array platform according to the manufacturer’s instructions (R & D systems). The data were processed using Bio-Plex manager software version 6 (Bio-Rad) with a five-parametric curve fitting. The intraplate coefficient of variation (CV) ranged from 7.0% to 12%, whereas the interplate CV was <14%. Samples were measured using reagents from the same lot to avoid lot-to-lot variations.

### Cell cultures, plasmids and transfections

Since ANGPTL3 protein is produced almost exclusively from the liver, a liver cell line (HepG2) was used throughout this study and cell line was obtained from American Type Culture Collection (Rockville, Baltimore, MD). Cells were cultured in Eagle’s Minimum Essential Medium supplemented with 10% fetal bovine serum and penicillin/streptomycin. For transient transfection assays, cells (at ~80% confluence) were transfected with 20 μg of DNA using the lipofectamine method as recommended by the manufacturer (Invitrogen, Carlsbad, CA).

### Western blot analysis

Western blots were carried out on whole peripheral blood mononuclear cell extracts or cell extracts prepared in RIPA buffer (50 mM Tris-HCl pH 7.5, 150 mM NaCl, 1% Triton x100, 1 mM EDTA, 0.5% sodium deoxycholate and 0.1% SDS). Protein concentration was determined by the Bradford method using globulin as a standard. After 20 μg of protein was resolved on 10% SDS-PAGE gels, the proteins were transferred onto PVDF membranes, blocked with 5% non-fat dried milk in Tris-buffered saline containing 0.05% Tween 20 for 1 h at room temperature and then probed with the primary antibody overnight at 4 °C. After washing, the membranes were incubated with horseradish peroxidase-conjugated secondary antibody for 2 h at room temperature and finally, protein bands were visualised by chemiluminescence. The images were captured using the Versadoc 5000 system (Bio-Rad). The primary antibodies used in this study were raised against ANGPTL3 (cat. ABC83, Millipore) and GAPDH (Millipore, Temecula, CA) was used as an internal control. For densitometric analysis, the intensity of the bands was determined using Quantity One Software (Bio-Rad).

### Luciferase binding assay

Luc-Pair Duo-Luciferase Assay Kit 2.0 (GeneCopoeia) was used to validate predicted miRNA targets on 3′-UTRs. The experiment included cotransfection of 3′-UTR clones (ANGPTL3/pCMV6) with miRNA (miR-181d/miR-CMV) in HEK293 and HepG2 cells. The luciferase assay was done 24 h posttransfection. The cells were lysed using the lysis buffer provided in the kit. The assay reagents and protocol used were as described in the kit manual. This assay detects and measures firefly luciferase (FLuc) and renilla luciferase (Rluc) sequentially, thus measuring the inhibitory effect of an miRNA on a target sequence. Luciferase LPL assays.

### LPL assays

To understand the effect of MIR-181d on LPL activity, HepG2 cells were transfected with MIR-181d, and compared with its control (empty vector). The activity was tested 48 hours post transfection. An LPL assay kit (Lipoprotein Lipase Activity Assay kit, Cell Biolabs Inc. Cat. No. STA-610-T) was used to quantitatively measure LPL activity. The assay was performed based on the kit protocol. Briefly, samples and standards were added to a 96-well fluorescence microtitre plate. Then, diluted LPL fluorometric substrate was added to each well. The plate was protected from light and incubated for 30 mins at 370 C. The reaction was stopped, and the fluorescence was read in a microplate reader at excitation 480–485 nm range and emission 515–525 nm range. The LPL activity was calculated comparing the sample fluorescence to the standard curve. activity was determined using a plate luminometer (SynergyH4).

### Statistical analysis

Statistical analysis was performed with SAS version 9.2 (SAS Institute Inc., Cary, NC). Unless otherwise stated, all descriptive statistics for the variables were reported as means ± standard error of the mean (SEM). A t-test was used to determine the significance of differences in means between the two groups as indicated in the figure legends. Correlations between variables were calculated with Spearman’s rank correlation test. Differences were considered statistically significant at *P* values less than 0.05.

## Results

### Characteristics of the study population

The physical characteristics of the two groups enrolled in the study are displayed in Table 2. As expected, BMI was significantly higher in the obese group compared with the non-obese group (*P* < *0*.*001*). The obese group was significantly older than the non-obese group. The obese group had lower HDL and higher TG levels (*P* = *0*.*019* and *P* = *0*.*057*, respectively). Compared with the non-obese group, the glucose and HbA1C levels were significantly higher in the obese group (*P* < *0*.*01*).

**Table 2:** Characteristics of study population.

Characteristics	Non-obese Average ± SEM (N = 82)	Obese Average ± SEM (N = 62)	*p*-value
Age (Year)	44.225 ± 1.038	48.319 ± 1.043	**0.006**
Weight (KG)	70.442 ± 0.971	94.148 ± 1.14	**<0.001**
Height (M)	1.649 ± 0.008	1.655 ± 0.008	0.556
BMI (KG/M^2^)	25.806 ± 0.251	34.255 ± 0.259	**<0.001**
WCHipR	0.866 ± 0.010	0.942 ± 0.018	**<0.001**
Chol (mmol/l)	5.001 ± 0.098	5.053 ± 0.09	0.701
HDL (mmol/l)	1.334 ± 0.043	1.209 ± 0.031	**0.02**
LDL (mmol/l)	3.096 ± 0.086	3.158 ± 0.084	0.604
TGL (mmol/l)	1.274 ± 0.085	1.516 ± 0.086	**0.047**
GLU (mmol/l)	5.904 ± 0.164	7.313 ± 0.267	**<0.001**

### Human ANGPTL3 mRNA is a direct target of miR-181d

**Figure 1:**
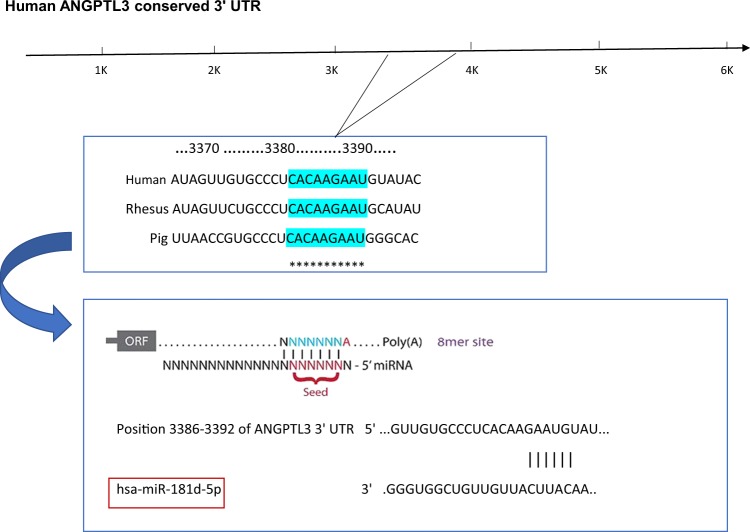
miRNA bioinformatics prediction analyses for 3′UTR site of ANGPTL3 gene highlighting the conserved binding site for miR-181d molecule.

Targetscan and miRWalk prediction software were used to identify the target genes of miR-181d, which allowed us to aggregate and compare results from other miRNA-to-mRNA databases. *ANGPTL3* was identified as a possible target of miR-181d. Figure 1 shows a predicted binding site for miR-181d in the 3′-UTR of human *ANGPTL3* mRNA. To further confirm the direct interaction between miR-181d and the 3′-UTR of *ANGPTL3* mRNA, overexpression and dual-luciferase reporter binding assay were performed.

### Reduced expression of miR-181d in plasma and adipose tissue of obese humans

**Figure 2:**
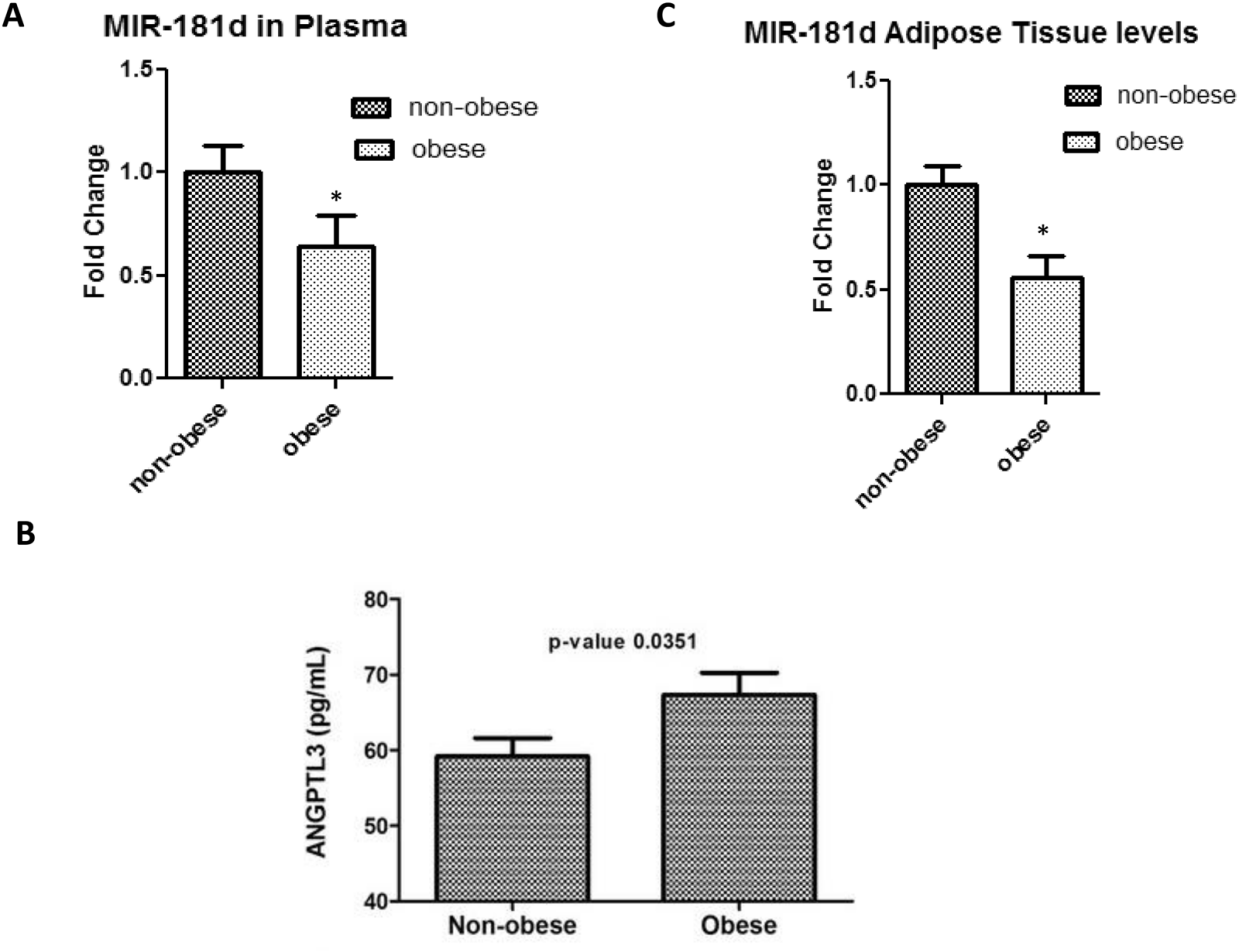
Gene expression analyses for both ANGPTL3 and miR-181d in obese and non-obese humans using both plasma and adipose tissue.

To assess the expression of miR-181d in obesity, relative gene expression analysis was done on plasma samples from our 144 subjects. miR-181d expression was significantly higher in non-obese subjects (0.98 ± 0.13) compared with the obese individuals (0.64 ± 0.15, *P* = 0.045, Fig. 2A). By contrast, ANGPTL3 was higher in the obese than in the non-obese group (Fig. 1B).

To validate the data on gene expression observed in the plasma, we performed real-time PCR analysis of miR-181d using RNA from adipose tissue (n = 10 for each group). Consistent with the plasma data, *mIR*-*181d* was more than 1.5-fold lower in the obese compared with the non-obese group (Fig. 2C). Taken together, these results indicate that obesity is associated with a significant reduction in the expression of miR-181d in both plasma and adipose tissue.

### Confirmation of binding between miR-181d and ANGPTL3 gene

**Figure 3:**
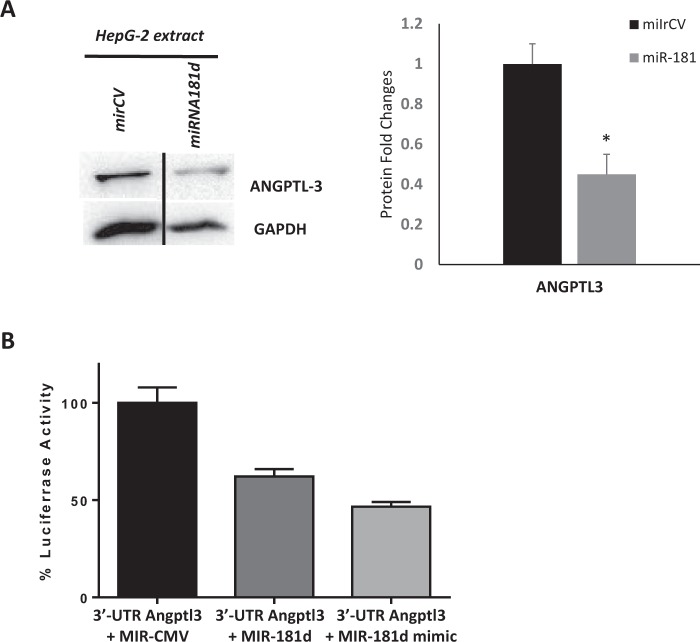
miR-181d binding and repression to ANGPTL3 protein. (**A**) Western Blot image of protein from HepG2 cells that were overexpressed with miR-181d and its effect on the expression of ANGPTL3. (**B**) Luciferase binding assays between miR-181d and ANGPTL3 5-UTR.

To confirm binding between miR-181d and the *ANGPTL3* gene, overexpression and luciferase binding techniques were performed using a specific miR-181d clone. Overexpression of miR-181d in HepG2 cells resulted in reduced levels of ANGPTL3 protein compared with those in control cells (Fig. 3A). A luciferase reporter binding assay containing either wild-type or control-UTR of *ANGPTL3* was used. Transfection of the miR-181d clone or miR-181d mimic indeed significantly reduced luciferase expression from the wild-type, and but not the control reporter plasmid (50% reduction, Fig. 3B). In agreement with the inverse association between miR-181d and ANGPTL3 observed in the previous experiments, miR-181d seemed to selectively bind and repress ANGPTL3 protein.

### Correlation analysis of miR-181d with clinical profiles, inflammatory and metabolic stress markers

**Figure 4:**
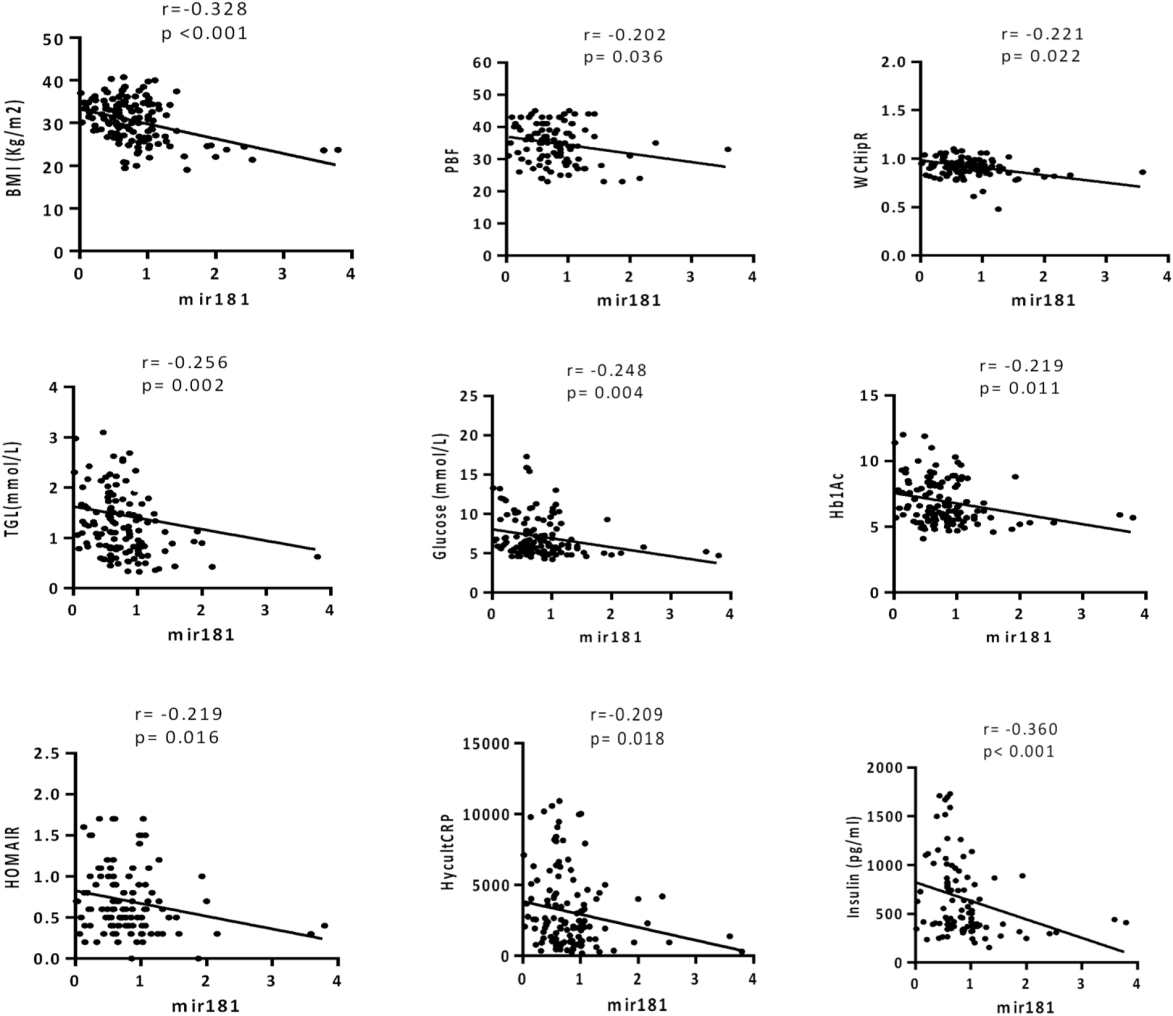
Down regulation of ANGPTL3 protein following *in vitro* co-transfection with miR-181d in HepG2 cell line following PA treatment.

To understand the physiological consequences of the reduction of miR-181d in obese individuals, the subjects’ clinical profile and inflammatory and metabolic stress responses were investigated for a correlation with miR-181d levels using Spearman’s rank test. There was a negative correlation between the levels of miR-181d and indicators of obesity such as BMI (r^2^ = −0.328; *P* < *0*.*001*) and percentage of body fat (r^2^ = −0.202; *P* = *0*.*036*; Fig. 4). Negative correlations were also found with TG levels (r^2^ = −0.256; *P* = *0*.*002*) and WCHip (r^2^ = −0.221; *P* = *0*.*022*). There were also significant negative associations with glucose (r^2^ = −0.248; *P* = *0*.*004*), Hb1Ac (r^2^ = −0.219; *P* = *0*.*011*), insulin (r^2^ = −0.360; *P* *<* *0*.*001*), hsCRP (r^2^ = −0.209; *P* = *0*.*018*) and HOMA-IR (r^2^ = −0.219; *P* = *0*.*016*). No correlation was found among the other parameters (Table 3; some data not shown).

**Table 3:** Correlation analysis for miR-181d with clinical and lipid biomarkers.

Phenotype	Correlation Coefficient	p-value^#^	Correlation Coefficient*	p-value*
Age	−0.15	0.077		
BMI (kg/m^2^)	−0.328	**<0.001**	−0.308	**<0.001**
PBF	−0.202	**0.036**	−0.234	**0.016**
WCHipR	−0.221	**0.022**	−0.247	**0.011**
Chol (mmol/l)	−0.005	0.957	−0.002	0.981
HDL (mmol/l)	0.144	0.094	0.167	0.053
LDL (mmol/l)	−0.049	0.57	−0.061	0.486
TGL (mmol/l)	−0.256	**0.002**	−0.237	**0.006**
GLU (mmol/l)	−0.248	**0.004**	−0.211	**0.014**
HBA1C (%)	−0.219	**0.011**	−0.176	**0.044**
WBC	−0.202	**0.019**	−0.195	**0.025**
Insulin U/L	−0.118	0.259	−0.097	0.359
Hycult CRP	−0.209	**0.018**	−0.191	**0.033**
HOMA-IR	−0.219	**0.016**	−0.195	**0.034**
HOM-Ab	0.133	0.149	0.100	0.280

^#^Non-parametric spearman correlation test, *Non-parametric partial correlation test adjusting for age and gender.

### miR-181d selectively binds to and represses ANGPTL3 in HepG2 cells

**Figure 5:**
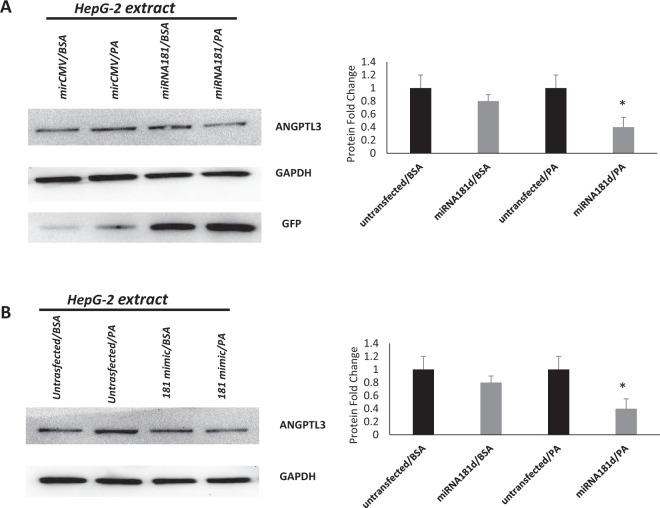
Negative correlation between miR-181d, obesity index and ANGPTL3 levels in the circulation.

ANGPTL3 has been previously reported to regulate lipid metabolism by inhibiting LPL and therefore TG clearance, and miR-181d can reduce cellular TG and cholesterol ester and will likely have an effect opposite to that of ANGPTL3. To test this possibility, the HepG2 cell line was treated with palmitic acid, followed by transfection with miR-181d. Palmitic acid treatment resulted in a significant increase in endogenous ANGPTL3 protein expression compared with untreated control cells. Following miR-181d transfection, the expression of ANGPTL3 was significantly reduced compared with the control (no mIR-181d, Fig. 5A,B), supporting the anti-obesity role of miR-181d through binding and inhibition of ANGPTL3 expression.

### miR-181d inhibits ANGPTL3 gene and increases LPL activity

**Figure 6:**
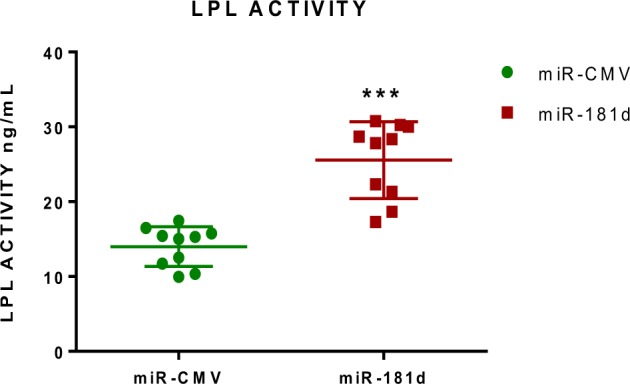
LPL activity from cell lysate of HepG2 transfected with miR- 181d and its control. A significant increase was observed in cells transfected with miR-181d as compared to its control (P > 0.0001).

As a readout for the ANGPTL3 inhibition by miR-181d, we sought to overexpress miR-181d in HepG2 liver cell and assess the LPL activity. Interestingly, our result showed a significant increase in LPL activity in the cell overexpressing miR-181d suggesting ANGPTL3 inhibition and restoration of LPL activity by miR-181d (Fig. 6).

## Discussion

This study was designed to evaluate the expression of miR-181d in obesity and to shed light on its role in lipid metabolism by modulating an important lipid-regulating protein, ANGPTL3. We report here for the first-time decreased expression of miR-181d along with increased expression of ANGPTL3 in obese people. *In vitro* overexpression of miR-181d molecules resulted in significant suppression of ANGPTL3 expression. Furthermore, luciferase binding assay using the 3′-UTR target regions of ANGPTL3 showed strong binding between miR-181d and ANGPTL3, suggesting a direct regulatory role for miR-181d on ANGPTL3. Taken together, our data support the protective role that miR-181d may play against obesity by reducing ANGPTL3 gene expression.

The miR-181 family is highly enriched in white adipose tissue and plays an instrumental role in controlling lipid metabolism by attenuating genes involved in lipid synthesis and increasing expression of genes involved in β-oxidation. Chu *et al*. reported that miR-181a was able to repress IDH1, a metabolic enzyme in the TCA cycle and subsequently modulated genes involved in lipid synthesis and β-oxidation, resulting in the inhibition of lipid accumulation^10^. Whittaker *et al*. reported that miR-181d plays a key role in regulating the lipid content of hepatocytes by targeting *Adamts 5*, a gene expressed in subcutaneous and gonadal adipose tissue and highly expressed in obesity^9^. They showed that miR-181d was the most effective inhibitor of hepatic lipid droplets, decreasing them by about 60%^9^. The same group also showed that miR-181d decreased cellular TG and cholesterol ester in various assays. Our findings indicate for the first time that miR-181d levels are decreased in the circulation and adipose tissue of obese human subjects, suggesting that higher levels may protect against obesity.

ANGPTL3 is one of the key regulators of plasma lipoproteins by virtue of its ability to inhibit LPL activity^15,22,32^. We previously showed that ANGPTL3 levels are elevated in obesity and diabetes^33^. ANGPTL3 is produced by the liver and then proteolytically cleaved by proprotein convertases to generate an active N-terminal domain that inhibits LPL activity through its interaction with ANGPTL8^22^. Earlier research revealed that loss of function mutations identified in ANGPTL3 were associated with reduced levels of VLDL, LDL, HDL and TG^23^. A significant negative correlation between ANGPTL3 blood levels and miR-181d was identified in our study cohort. Direct binding between miR-181d and ANGPTL3 was confirmed through a series of overexpression and luciferase binding assays. The findings support both the regulatory and protective roles of miR-181d against obesity by modulating ANGPTL3 expression.

**Figure 7:**
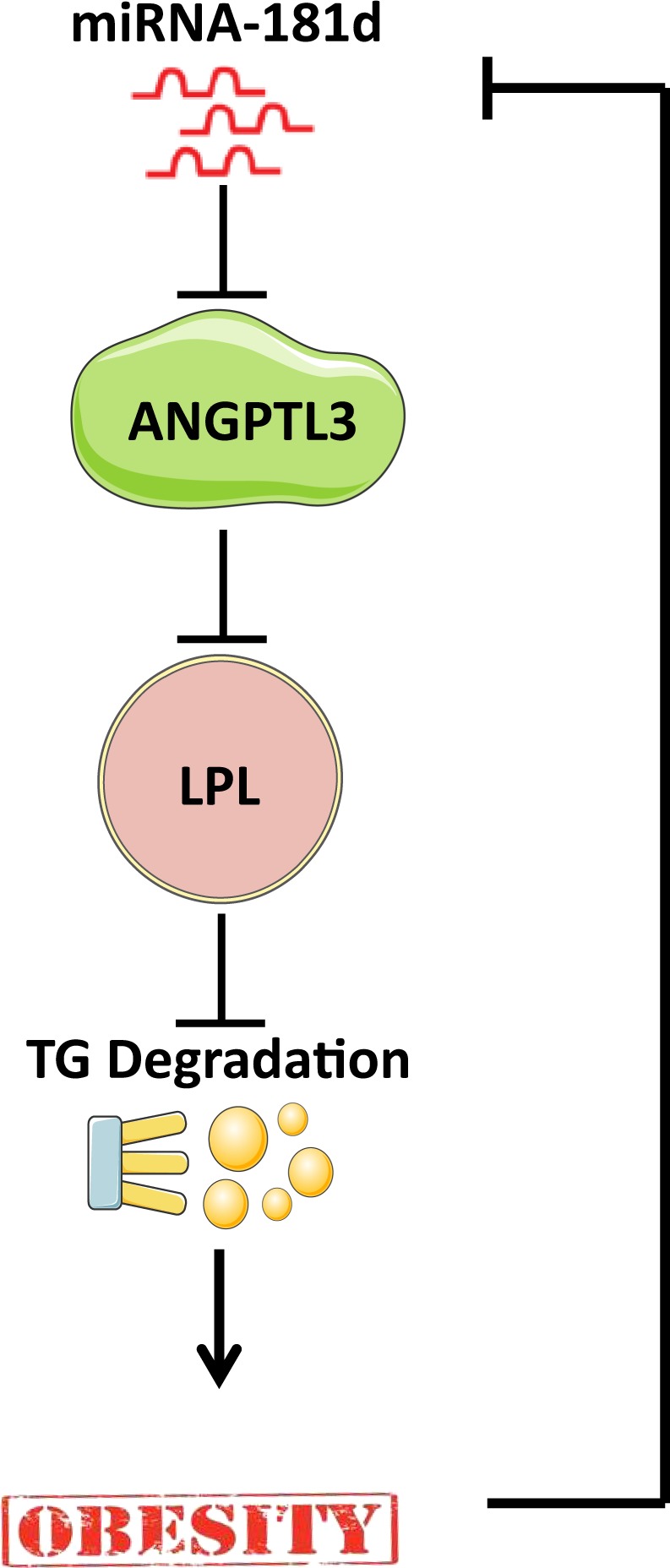
Proposed model for potential mechanisms for miR-181d Lipid metabolism regulation.

In the current study, miR-181d plasma levels were inversely correlated with TG levels, and ANGPTL3 levels were positively correlated with TG levels. Our *in vitro* cell line experiments clearly showed that overexpression of miR-181d led to major repression of ANGPTL3 protein expression. ANGPTL3 is known to inhibit the activity of LPL, which is a key enzyme in the lipoprotein lipolysis pathway and TG degradation. Our data also showed that treatment with a free fatty acid significantly induced ANGPTL3 expression in a liver cell line, but overexpression of miR-181d significantly mitigated that induction of ANGPTL3. Based on this novel finding, we postulate that miR-181d overexpression represses ANGPTL3 protein activity and restores LPL activity to stop the lipoprotein lipolysis pathway, TG degradation and generation of free fatty acids. A proposed model for the effects of miR-181d and the mechanisms involved in the regulation of lipid metabolism through ANGPTL3 is suggested (Fig. 7).

One of the main limitations of this study is the cross-sectional design which did not allow us to establish causality regarding the roles that miR-181d and ANGPTL3 may therefore play in the development of obesity. However, the atherogenic role of miR-181d and ANGPTL3 is inferred from their function and what has been documented about their role in modulating plasma lipid content. Nonetheless, a prospective study will be better fit to answer this question and establish the role of these substances in obesity.

In conclusion, we have provided compelling evidence that the expression of miR-181d is markedly reduced in both blood and adipose tissue of obese humans. We further confirmed direct binding of miR-181d to ANGPTL3 and subsequent repression of this important protein that regulates lipid metabolism. The inverse relationship between miR-181d and ANGPTL3 and the opposing roles of these molecules in lipid metabolism support the concept that miR-181d has a protective role against obesity. Collectively, these findings reveal a novel miR-181d–ANGPTL3 axis that has a vital role in regulating lipid metabolism, and they suggest a potential role for miR-181d in therapy targeting dysregulated lipid metabolism in metabolic diseases.
